# Far red/near infrared light-induced protection against cardiac ischemia and reperfusion injury remains intact under diabetic conditions and is independent of nitric oxide synthase

**DOI:** 10.3389/fphys.2014.00305

**Published:** 2014-08-22

**Authors:** Agnes Keszler, Garth Brandal, Shelley Baumgardt, Zhi-Dong Ge, Phillip F. Pratt, Matthias L. Riess, Martin Bienengraeber

**Affiliations:** ^1^Department of Anesthesiology, Medical College of WisconsinMilwaukee, WI, USA; ^2^Department of Anesthesiology, Clement J. Zablocki VA Medical CenterMilwaukee, WI, USA; ^3^Department of Physiology, Medical College of WisconsinMilwaukee, WI, USA; ^4^Department of Pharmacology and Toxicology, Medical College of WisconsinMilwaukee, WI, USA

**Keywords:** near infrared light, nitrite reductase, ischemia and reperfusion injury, myoglobin, cardioprotection

## Abstract

Far red/near-infrared light (NIR) promotes a wide range of biological effects including tissue protection but whether and how NIR is capable of acutely protecting myocardium against ischemia and reperfusion injury *in vivo* is not fully elucidated. Our previous work indicates that NIR exposure immediately before and during early reperfusion protects the myocardium against infarction through mechanisms that are nitric oxide (NO)-dependent. Here we tested the hypothesis that NIR elicits protection in a diabetic mouse model where other cardioprotective interventions such as pre- and postconditioning fail, and that the protection is independent of nitric oxide synthase (NOS). NIR reduced infarct size dose dependently. Importantly, NIR-induced protection was preserved in a diabetic mouse model (db/db) and during acute hyperglycemia, as well as in endothelial NOS^−/−^ mice and in wild type mice treated with NOS inhibitor L-NAME. In *in vitro* experiments NIR light liberates NO from nitrosyl hemoglobin (HbNO) and nitrosyl myoglobin (MbNO) in a wavelength-(660-830 nm) and dose-dependent manner. Irradiation at 660 nm yields the highest release of NO, while at longer wavelengths a dramatic decrease of NO release can be observed. Similar wavelength dependence was observed for the protection of mice against cardiac ischemia and reperfusion injury *in vivo*. NIR-induced NO release from deoxymyoglobin in the presence of nitrite mildly inhibits respiration of isolated mitochondria after hypoxia. In summary, NIR applied during reperfusion protects the myocardium against infarction in an NO-dependent, but NOS-independent mechanisms, whereby mitochondria may be a target of NO released by NIR, leading to reduced reactive oxygen species generation during reperfusion. This unique mechanism preserves protection even during diabetes where other protective strategies fail.

## Introduction

Restoration of blood flow to a region of previously ischemic myocardium (reperfusion) is a critical life-saving intervention against tissue necrosis, but reperfusion itself also results in significant damage to the myocardium. Many therapeutic strategies such as ischemic and volatile anesthetic pre- and postconditioning have been developed and are effective in healthy animal models but few have translated successfully to patients (Ludman et al., 2010). A major reason for the resistance to cardioprotection against infarction by physical or pharmacological stimuli is the advanced age and/or presence of comorbidities such as diabetes in patients. For example, endothelial dysfunction appears to contribute to the lack of protection by ischemic or anesthetic postconditioing in diabetes (Raphael et al., 2010; Przyklenk et al., 2011). Activation of endothelial nitric oxide synthase (eNOS) and pro-survival signaling pathways, together with alteration of mitochondrial bioenergetics, contribute to the mechanisms of various cardioprotective strategies against ischemia and reperfusion injury (Tsang et al., 2004; Mio et al., 2009; Ge et al., 2010). Although nitric oxide synthases (NOS) produce a large part of endogenous nitric oxide (NO), there is considerable interest in NOS-independent generation of NO *in vivo*, particularly during hypoxia or anoxia, where low oxygen tensions limit NOS activity (Godecke, 2006; Hendgen-Cotta et al., 2008, 2010). Interventions that can increase NO bioavailability have significant therapeutic potential. Under hypoxic conditions, heme-containing proteins such as myoglobin (Mb) and hemoglobin (Hb) exhibit nitrite reductase activity which results in an increase in NO bound to the heme iron of Mb and Hb (Gladwin et al., 2006; Hendgen-Cotta et al., 2008). We have recently found that far red/near infrared light (NIR) both in purified systems and in myocardium can release NO from nitrosyl hemes (Lohr et al., 2009). Further, NIR protected cardiomyocytes and the rabbit heart from hypoxia and reoxygenation injury in a NO-dependent manner, reversible by NO scavenger cPTIO, and enhanced the protective effect of nitrite against ischemia and reperfusion injury of the rabbit heart (Lohr et al., 2009; Zhang et al., 2009).

NIR modulates biochemical systems by activating light-sensitive proteins harboring NIR-sensitive chromophores (Karu, 1999; Desmet et al., 2006). Previous studies suggested that NIR promotes cell survival during physiologic stress (Eells et al., 2003; Liang et al., 2008; Zhang et al., 2009). Repeated photostimulation of the myocardium has been demonstrated to be beneficial against long-term reperfusion injury in the rat and dog (Oron et al., 2001). For example, low-energy infrared (803 nm) laser irradiation delivered to the epicardium was shown to reduce scar formation and myocardial infarct size several weeks after prolonged coronary artery occlusion in dogs and rats (Oron et al., 2001). Aside from the heart, the beneficial effects of NIR light treatment have been studied in particular in a model of traumatic brain injury as well as in wound healing (Ankri et al., 2010; Naeser et al., 2011). NIR light treatment also improved the collateral blood vessel grow in a mouse model (tight skin mouse) of scleroderma (Zaidi et al., 2013). Frequently, the beneficial effects of NIR treatment have been associated with the stimulation of mitochondrial metabolism, particularly at the level of cytochrome c oxidase, complex IV of the electron transport chain (Karu, 2008). However, in a model of cardiac ischemia and reperfusion injury it is difficult to perceive how acceleration of cytochrome c oxidase at the time of reperfusion conveys protection to the heart. Rather, a mild reversible inhibition of the electron transport chain has been shown to reduce reactive oxygen species production during reperfusion, thereby increasing cardiomyocyte survival (Burwell et al., 2009). NO inhibits electron transport through competitive binding at complex IV and S-nitrosation at complex I (Piantadosi, 2012; Chouchani et al., 2013). Thus, we tested the hypothesis that brief exposure to NIR light at the time of reperfusion protects the heart in a wave length-dependent manner; and that this wave length dependence is paralleled by the release of NO from nitrosyl-heme proteins. We also examined whether NIR induced protection is maintained in a mouse model of acute hyperglycemia and diabetes (db/db) where protection by volatile anesthetics fail.

## Materials and methods

All experimental procedures and protocols used in this investigation were reviewed and approved by the Animal Care and Use Committee of the Medical College of Wisconsin. Furthermore, all conformed to the *Guiding Principles in the Care and Use of Animals* of the American Physiologic Society and were in accordance with the *Guide for the Care and Use of Laboratory Animals*.

### Myocardial ischemia and reperfusion injury in mice

A murine model of myocardial ischemia and reperfusion injury was used as previously described (Ge et al., 2010). C57Bl/6 (wild type) mice, as well as eNOS^−/−^ and diabetic db/db mice were used for these experiments. Glucose (2 g/kg) was administered intraperitoneal 10 min before ischemia to produce hyperglycemia. Mice were anesthetized by intraperitoneal injection of sodium pentobarbital (100 mg/kg) and ventilated with room air supplemented with 100 % oxygen at a rate of 100 breaths/min with a tidal volume of approximately 0.25 ml using a rodent ventilator (Harvard Apparatus, South Natick, MA). Body temperature was maintained between 36.8 °C and 37.5 °C. Myocardial ischemia was produced by occluding the left coronary artery (LAD) for 30 min, and reperfusion was initiated by loosening the suture and continued for 3 h.

### Experimental protocol

Mice were randomly assigned to receive no irradiation (control) or NIR irradiation applied to the epicardial surface (670 nm, 170 mW/cm^2^) with an LED array (NIR Products LLC, Milwaukee, WI) for 1 min before and through the first 4 min of reperfusion (energy-density equivalent to 51 J/cm^2^). Separate experiments were performed to evaluate the energy- and wavelength-dependence of NIR-mediated cardioprotection by varying the array (670, 740, 830 nm) and the output of the device.

C57BL/6, eNOS^−/−^ mice and db/db mice were used to explore the dependence of NIR-mediated cardioprotection on eNOS and its efficacy in a diabetic animal.Acute hyperglycemia was induced by administration of D-glucose (2 g/kg) in C57BL/6 mice 10 min before ischemia. Mannitol (1.82 g/kg) was used for osmotic control in preliminary experiments in C57BL/6 mice both with and without NIR treatment and did not exhibit any significant effect compared to mice that did not receive mannitol. Pharmacological inhibition of eNOS was used to complement the experiments in eNOS^−/−^ mice and thus, C57BL/6 mice received 1mg/kg, i.v. of the non-selective NOS inhibitor L-NAME prior to LAD occlusion and reperfusion.

### Determination of myocardial infarct size

For infarct size measurements, the heart was first stained by cannulation of the aorta with a 1 % solution of 2,3,5-triphenytetrazolium chloride. Then the suture previously placed around the left descending coronary artery was retied and diluted phthalo blue dye was injected through the same cannula. As a result of these procedures, the non-ischemic portion of the left ventricle was stained dark blue. Viable myocardium within the area at risk was stained bright red, and infarcted tissue was light yellow. The heart was then excised and ventricles were cut into 4–5 uniform transverse slices of 2 mm thickness using a mouse heart matrix. Slices were then analyzed by planimetry.

### Nitrosyl hemoglobin (HbNO) and nitrosyl myoglobin (MbNO) preparation

Oxyhemoglobin purified from human blood according to a published procedure (Rossi-Fanelli et al., 1961) was deoxygenated, or solution of metmyoglobin (from horse skeletal muscle, Sigma) was reduced in an anaerobic chamber with Na_2_S_2_O_4_ in phosphate buffered saline (PBS, pH 7.4). Then the heme proteins were nitrosylated by addition of equivalent concentration of highly concentrated PROLI NONOate (Cayman Chemicals, Ann Arbor, Mi) dissolved in 0.1 N NaOH. The process was spectrophotometrically followed. Solutions were made daily, and used immediately.

### NO-dependent chemiluminescence analysis

A Sievers 280i Nitric Oxide Analyzer (General Electric, Boulder, CO) was used to detect NO evolved from nitrosyl species as a consequence of NIR irradiation. HbNO or MbNO (3 ml of 10 μM) was placed into the purge vessel of the analyzer, and externally irradiated at various powers and wavelengths for 1 min. Detector response for NO liberated from known amounts of PROLI NONOate injected into PBS pH 7.4 was used as a basis of quantification.

### Measurement of oxygen consumption in isolated mitochondria

Rat heart mitochondria were isolated by differential centrifugation as previously reported (Pravdic et al., 2010). Mitochondrial oxygen consumption was measured with a Clark-type oxygen electrode (Hansatech Instruments, Norfolk, UK) at 30 °C in respiration buffer containing mitochondria at a final concentration of 1 mg protein/mL. The mitochondrial respiration buffer was composed of 130 mM KCl, 5 mM KH_2_PO_4_, 20 mM MOPS, 2.5 mM EGTA, 1 mM Na_4_P_2_O_7_, and 0.1 % BSA, at pH 7.4. State 2 respiration was initiated with 5 mM pyruvate and 5 mM malate as substrates. The adenosine diphosphate (ADP)-stimulated oxygen consumption (state 3 respiration) was measured in the presence of 250 μM ADP. After hypoxia was reached mitochondria were incubated with deoxymyoglobin (40 μM; prepared from myoglobin with sodium dithionite as reducing agent) and sodium nitrite (20 μM) for 1 min and then exposed directly to NIR (170 mW/cm^2^) for another min. After that the chamber was opened to allow reoxygenation. A faster rate of reoxygenation of the chamber indicated an inhibition of respiration.

### Statistical analysis

Statistical analysis of data within and between groups was performed with analysis of variance (ANOVA) for repeated measures followed by the Student-Newman-Keuls test. Changes were considered statistically significant when *P* < 0.05. All data are expressed as mean ± standard deviation (SD) unless otherwise indicated.

## Results

**Figure 1:**
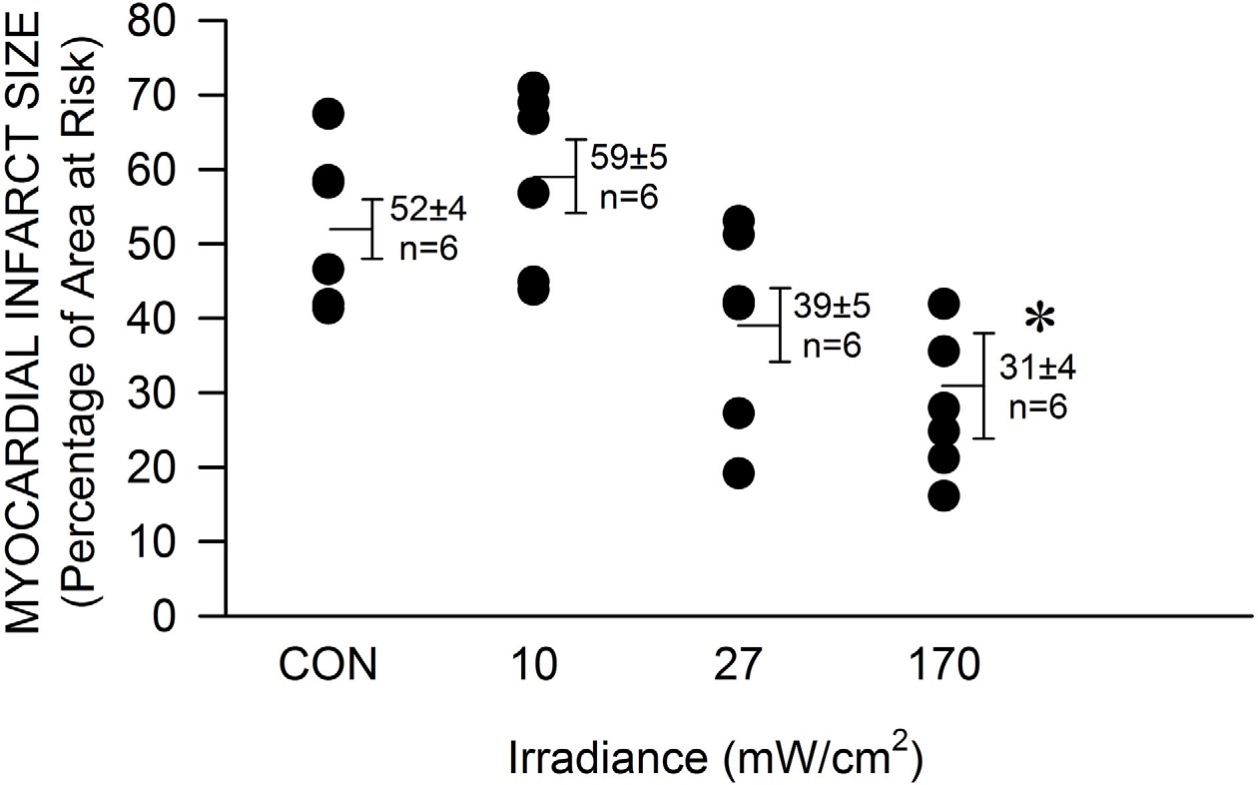
**The NIR-mediated cardioprotection is irradiance-dependent**. The exposed mouse heart was irradiated at 670 nm with various irradiances for 1 min during ischemia and 4 min during reperfusion. The lamp was placed 5 mm above the heart. At 170 mW/cm^2^ a significant decrease in infarct size was observed. Values are means ± SD, ^*^p < 0.05 compared to untreated control (CON).

The mouse was chosen as model to determine the efficacy of NIR-mediated protection against cardiac ischemia and reperfusion injury in order to expand our findings on NIR-induced protection in rabbits and due to the advantage of the large availability of genetically engineered animals. Exposure to NIR for the last min of occlusion and first 4 min of reperfusion significantly (*P* < 0.05) reduced infarct size at the highest chosen irradiance (170 mW/cm^2^, corresponding to 51 J/cm^2^) compared to control experiments without NIR exposure (31 ± 7 vs. 51 ± 4 % of left ventricular area of risk, Figure 1). The effect of NIR on infarct size was energy dependent. Myocardial infarct size was 59 ± 5 and 39 ± 6 %, at an irradiance of 10 and 27 mW/cm^2^, corresponding to 3 and 8.1 J/cm^2^ respectively. Thus, the threshold of cardioprotection appeared to occur at an irradiance level of 30 mW/cm^2^. Importantly, no increase in epicardial surface temperature upon exposure to NIR was observed.

**Figure 2:**
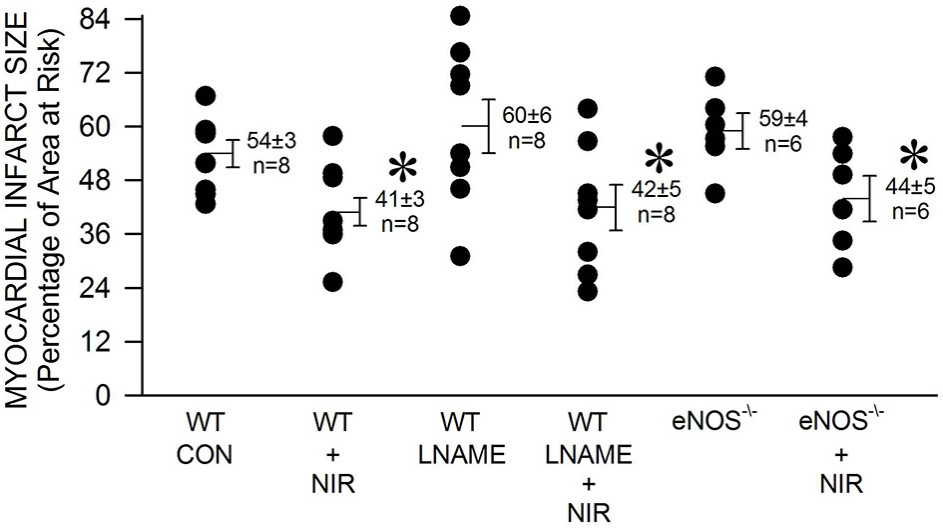
**The NIR-mediated cardioprotection is NOS-independent**. The exposed heart of wild type and eNOS^−/−^ mice was irradiated at 670 nm with 170 mW/cm^2^ power for 1 min during ischemia, and 4 min during reperfusion. Mice treated with L-NAME (10 mg/kg, administered 10 min prior occlusion) were also examined. Irradiation was equally protective in the case of control, L-NAME-treated, and eNOS^−/−^ mice. Values are means ± SD, ^*^p < 0.05 when compared to untreated control (CON).

We then tested whether NOS is involved in the mechanism of NIR-induced protection. Pretreatment with the non-selective NOS inhibitor L-NAME had no effect alone (60 ± 6 % infarct size of area at risk), nor did it inhibit NIR-mediated reduction in infarct size (42 ± 5 %). Similarly, eNOS^−/−^ mice were also protected against myocardial ischemia and reperfusion injury by NIR treatment (44 ± 5 % compared to 59 ± 4 % without treatment). An irradiance of 170 mW/cm^2^ was applied in all cases. These data suggest that NIR-mediated cardioprotection is independent of the activity of NOS (Figure 2).

**Figure 3:**
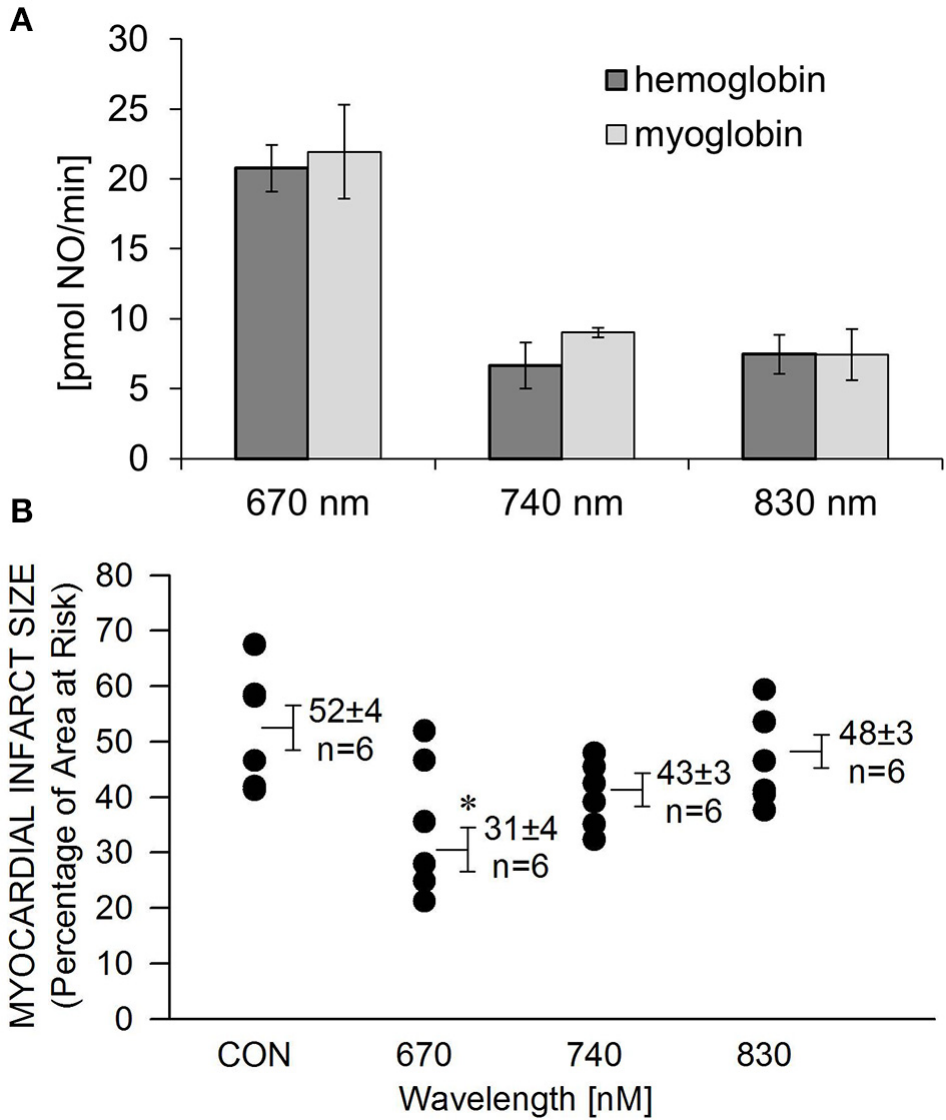
**Wavelength-dependence of NIR-induced NO release is in accordance with wavelength dependence of cardioprotection. (A)** A solution of HbNO or MbNO (10 μM in PBS, pH 7.4) was irradiated at various wavelengths with 10 mW/cm^2^ power for 1 min in the chamber of the NO analyzer. Formation of NO was on-line detected with ozone-based chemiluminescence. Maximum NO liberation was achieved at 660 nm irradiation with both heme nitrosyl compounds. Values are means ± SE, *n* = 3. **(B)** The exposed heart was irradiated at various wavelengths at170 mW/cm^2^ irradiance for 1 min during ischemia, and 4 min during reperfusion. Irradiation at 670 nm was the most cardioprotective. Values are means ± SD, ^*^p < 0.05 when compared to untreated control (CON).

We recently reported (Lohr et al., 2009) that NIR light has the capacity to liberate NO from nitrosylated hemoglobin (HbNO) and myoglobin (MbNO). Here we examined the wavelength dependence of NO release and protection. A solution of HbNO or MbNO (10 μM) was placed into the purge vessel of a chemiluminescence-based NO analyzer and subjected to irradiation for 1 min. We observed 2–3 times more NO released at 670 nm compared to 740 and 830 nm at 10 mW/cm^2^ irradiance (Figure 3A). As NIR does not have to penetrate tissue in these experiments, less irradiance compared to what is required for protection of the *in vivo* heart is needed. There was no significant difference between NO liberation from HbNO and MbNO. A similar trend was found with the wavelength dependence of NIR-induced reduction of infarct size. In contrast to 670 nm no significant protection was observed at 740 and 830 nm (Figure 3B).

**Figure 4:**
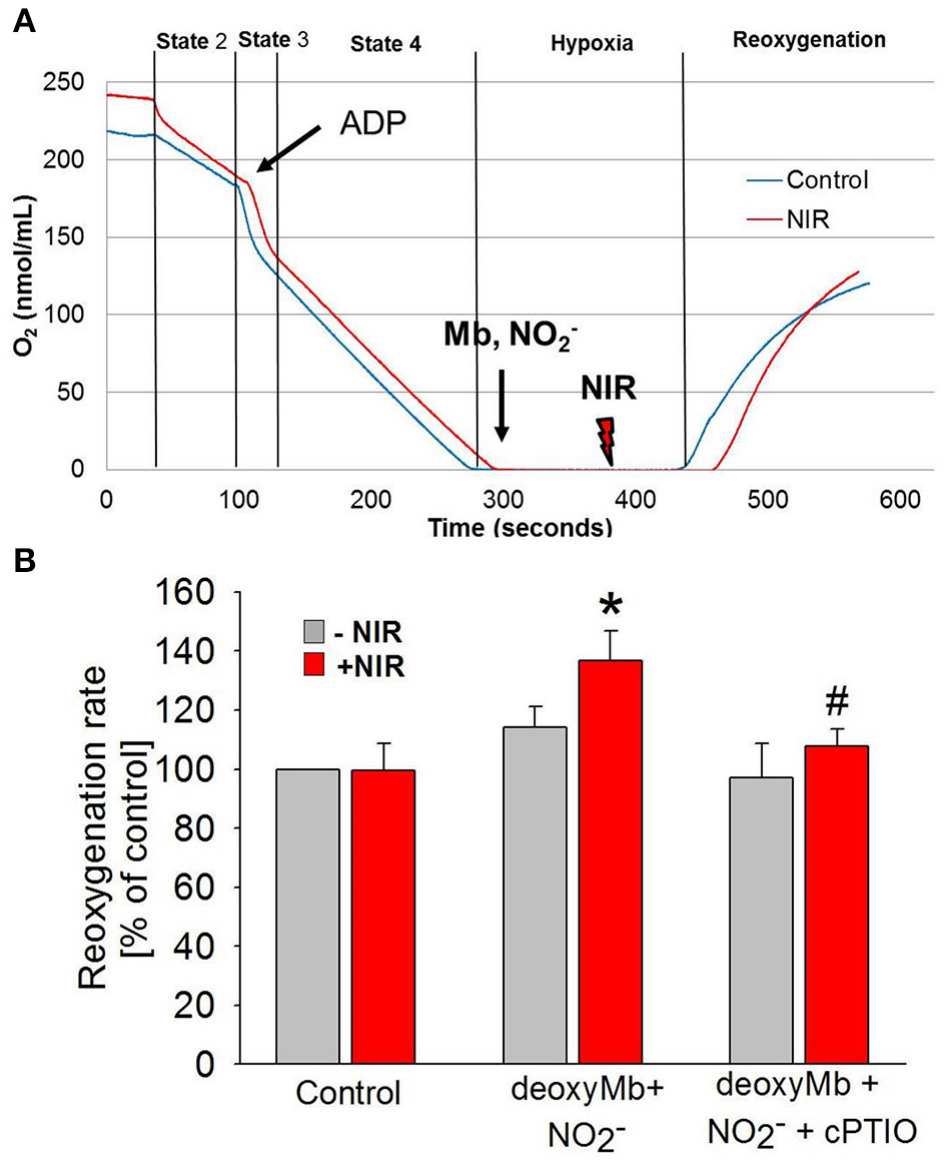
**NIR slows mitochondrial respiration in the presence of deoxymyoglobin and nitrite during reoxygenation. (A)** Oxygen consumption of purified cardiac mitochondria was monitored with a Clarke-type oxygen electrode. At the beginning of ischemia, deoxyMb and sodium nitrite were added. During the second minute of ischemia before reoxygenation, mitochondria were exposed to NIR at 670 nm with 50 mW/cm^2^ irradiance. **(B)** NIR caused an NO-dependent increase of reoxygenation rate (corresponding to a decrease of respiration) in the presence of deoxyMb and nitrite. The effect was reversed with NO scavenger CPTIO. Values are means ± SD, *n* = 6, ^*^p < 0.05 when compared to control, ^#^*p* < 0.05 when compared to NIR treated mitochondria in the absence of CPTIO.

Mitochondria are a potential therapeutic target of NO produced at the time of reoxygenation (Chouchani et al., 2013). Therefore, experiments were designed to establish the net outcome of NIR-enhanced nitrite reductase activity on mitochondrial respiration after hypoxia. We measured the reoxygenation rate of a mitochondrial suspension after hypoxia in the presence of deoxymyoglobin and nitrite, with and without NIR (660 nm, 50 mW/cm^2^) (Figure 4). While Mb and nitrite induce inhibition of mitochondrial respiration alone (Shiva et al., 2007a,b; Hendgen-Cotta et al., 2008), we hypothesized that light enhances this inhibition through its action on MbNO formed as a consequence of nitrite reductase activity of heme. We found that NIR, while alone had no considerable effect, could potentiate the inhibition caused by Mb and nitrite at lower nitrite doses. It triggered a significantly faster reoxygenation and thereby a decrease in respiration rate in the presence of deoxymyoglobin and nitrite than solely deoxymyoglobin and nitrite would induce. A partial compensatory effect of NO bound to and released from complex IV cannot be excluded, however, in the investigated *in vitro* system Mb was present in wide excess over cyt c oxidase (0.64 mg/ml Mb vs. 1 mg/ml total mitochondrial protein), and NIR was switched off at the time of reoxygenation. Thus, NO released from Mb might partially bind to cytochrome c oxidase or mediate S-nitrosation of complex I at the beginning of reoxygenation, thereby accelerating reoxygenation and inhibiting respiration. The observed effect is relevant since a mild reversible inhibition of the mitochondrial electron transport chain during cardiac reperfusion has been shown to reduce reactive oxygen species production. In the presence of NO scavenger cPTIO (10 μM), the NIR effect was reversed.

In diabetes, endothelial dysfunction, including defective NOS, is considered one of the causes for the failure of protective strategies such as ischemic or anesthetic pre- and postconditioning to reduce cardiac ischemia and reperfusion injury. Therefore, a NOS-independent mechanism of NO generation may allow NIR to reduce ischemia and reperfusion injury in the hyperglycemic or diabetic heart. Indeed, a similar degree of NIR-induced cardioprotection was observed in mice that were exposed to acute hyperglycemia (39 ± 4 % myocardial infarct size of area at risk vs. 52 ± 2 % without NIR) and in the diabetic db/db mouse (43 ± 4 vs. 61 ± 3 %) compared to the wild type mice (41 ± 3 % vs. 56 ± 3 %).

## Discussion

The current results demonstrate that a brief exposure to NIR immediately before and during early reperfusion protects the myocardium against infarction in an NOS-independent mechanism. Mitochondria are one potential therapeutic target of NIR-induced release of NO but other targets such as NO-sensitive guanylyl cyclase require further investigation. Importantly, NIR protects the hyperglycemic and diabetic heart. The absence of such protections has been one of the major hurdles in the implementation of pharmacological pre- and particularly postconditioning into the clinical setting.

In the nineteen nineties in Russia patients with coronary heart disease with prior myocardial infarction were exposed repeatedly to NIR by low-level laser therapy (LLLT) applied to the area of the heart on the skin. Lipid peroxidation was significantly reduced after NIR but little is known on whether cardiac function improved (Zubkova et al., 1993; Sorokina et al., 1997). In subsequent studies NIR was applied after chronic myocardial infarction in rat and dog models. NIR (803 nm, 6 mW/cm^2^ at the surface of the myocardium for 3 min, at 4-6 different locations) was applied twice, 15 min and 3 days after myocardial infarction, through the open chest directly onto the myocardium in dogs, and through the intercostal muscles in rats. Both mortality and infarct size were significantly reduced compare to untreated animals (Oron et al., 2001). Irradiation with NIR after myocardial infarction in rats resulted in a significant improved mitochondrial bioenergetics, and an increase in an inducible heat shock protein (HSP70), vascular endothelial growth factor (VEGF) and inducible nitric oxide synthase (iNOS) expression (Yaakobi et al., 2001). This was paralleled by a significant elevation in angiogenesis (Tuby et al., 2006). More recently, increased angiogenesis and collateralization upon NIR exposure (670 nm, 50 mW/cm^2^, 10 min per day for 14 days) with LED have also been reported in the ischemic hind limb of mice and rabbits (Lohr et al., 2013). In a mouse model for systemic sclerosis, an autoimmune connective tissue disorder characterized by oxidative stress, impaired vascular function, and attenuated angiogenesis, NIR stimulated angiogenesis by increasing angiomotin and decreasing angiostatin expression in the ischemic hind limb (Zaidi et al., 2013).

In the present study we found that myocardial infarction can be prevented from occurring, or at least reduced by a one-time NIR treatment right at the time of reoxygenation. It appears highly unlikely that NIR-induced reductions in infarct size were attributed to increased collateral perfusion as NIR-induced angiogenesis typically occurs as a result of sustained stimulation over several days. Therefore, the underlying mechanism of protection is likely different. Under hypoxic conditions heme-containing proteins such as myoglobin (Mb) and hemoglobin (Hb) exhibit nitrite reductase activity which results in an increase in NO liberation (Gladwin et al., 2006; Hendgen-Cotta et al., 2008). The NO formed may subsequently react with available deoxyHb or deoxyMb to yield iron-nitrosyl Hb (HbNO) or iron-nitrosyl Mb (MbNO). Thus, HbNO and MbNO may represent a significant storage pool of NO in the heart. Here we have demonstrated both for purified hemoglobin and myoglobin that NIR can decay nitrosyl heme and release NO in a wavelength-dependent manner. Importantly, the highest NO release was recorded at 670 nm where protection against ischemia and reperfusion injury was present (Figure 3). This further suggests a distinct mechanism from the previously reported protection through repeated NIR treatment in the permanently ligated heart where longer wavelengths were equally protective. We previously reported in the ischemic rabbit heart, after infusion of sodium nitrite, a large increase in nitrosyl heme formation as measured by electro paramagnetic resonance spectroscopy (EPR). The MbNO signal was reduced in the ischemic zone by NIR treatment suggesting dissociation of the heme-NO bond upon irradiation (Lohr et al., 2009).

Frequently, the beneficial effects of NIR treatment have been associated with the stimulation of mitochondrial metabolism, particularly at the level of cytochrome c oxidase, complex IV of the electron transport chain and concomitant enhancement of ATP synthesis (Karu, 2008). NIR may directly affect cytochrome c oxidase activity through one of its redox active metal centers. In addition, it has been suggested that NIR exerts its action on cytochrome c oxidase by a mechanism via NO release. The activated cytochrome c oxidase may not only cause changes in electron transport chain activity, including ROS generation, but released NO is available for other biological processes such as vasodilation and gene expression. However, compared to potential NO release from HbNO or MbNO the relative amounts of NO in the case of cyt c oxidase is limited (Osipov et al., 2007). Further, it is difficult to perceive how acceleration of cytochrome c oxidase at the time of reperfusion conveys protection to the heart. Rather, a mild reversible inhibition of the electron transport chain has been shown to reduce reactive oxygen species production during reperfusion and increase cardiomyocyte survival (Burwell et al., 2009). This was confirmed in ischemic isolated mitochondria where, in the presence of deoxmyoglobin and sodium nitrite, a decrease in respiration was detected upon reoxygenation of mitochondria after application of NIR (Figure 4). NO signaling may lead to S-nitrosation of a cysteine residue in complex I that has been implicated in protection against cardiac ischemia and reperfusion injury (Cochain et al., 2013). Reversible S-nitrosation of complex I slows the reactivation of mitochondria during the crucial first minutes of the reperfusion of ischemic tissue, thereby decreasing ROS production, oxidative damage and tissue necrosis.

**Figure 5:**
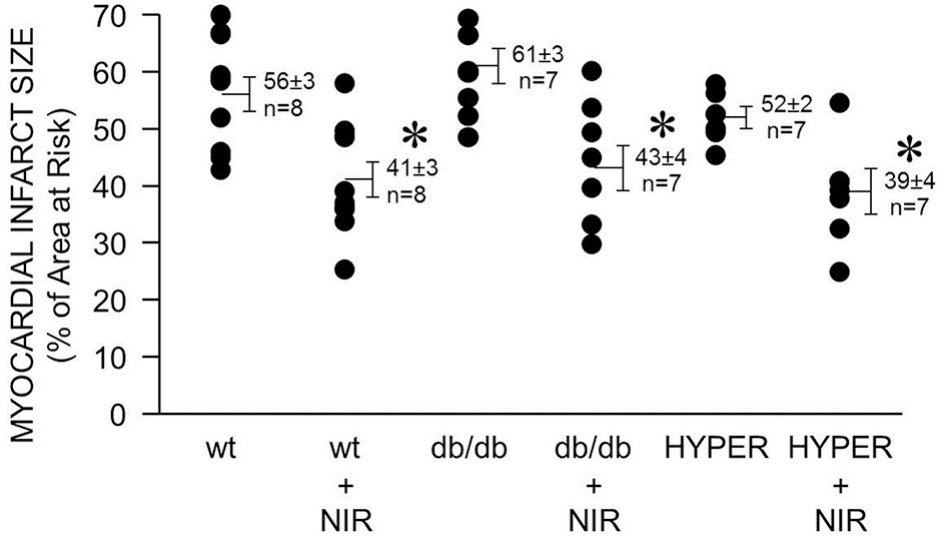
**NIR is cardioprotective in the presence of diabetes or hyperglycemia**. The exposed heart of wild type, db/db, and hyperglycemic mice was irradiated at 670 nm with 170 mW/cm^2^ irradiance for 1 min during ischemia, and 4 min during reperfusion. Light exposure resulted in a similarly effective decrease of infarct size in all three cases. Values are means ± SD, ^*^p < 0.05 when compared to untreated wild type.

Due to the reasonably high tissue penetration paralleled by limited potential of tissue damage NIR is attractive for the use in ischemic heart disease. While the required light power needs to be verified for human cardiac use, by comparing animal studies through various species and experimental settings an irradiance of 10–100 mW/cm^2^ for 2–10 min seems a reasonable starting point to achieve beneficial effects of NIR. A higher irradiance may be required for acute prevention of ischemia and reperfusion injury at the time of reperfusion. NIR treatment of the heart may be protective on patients after acute myocardial infarction or on ischemic heart conditions that are not accessible to current revascularization procedures. NIR could be particularly useful in the presence of comorbidities such as diabetes. Diabetes is an independent predictor of increased cardiovascular risk and myocardial infarct size is directly related to increases in blood glucose concentration in animals with or without diabetes (Van Der Horst et al., 2007). The mechanism of light-induced release of NO from iron-nitrosylated heme protein is likely to be maintained during diabetes and thus NIR may be protective from ischemia and reperfusion injury where other strategies such as ischemic and pharmacologic pre- and postconditioning fail (Kersten et al., 1998; Przyklenk et al., 2011). Indeed, we found that under acute hyperglycemia or in a mouse model of type 2 diabetes (db/db mouse) NIR exposure of the mouse heart at the time of reperfusion reduces infarct size significantly (Figure 5).

While there is substantial clinical promise for the use of NIR in heart disease several hurdles need to be considered and overcome. The technical challenge related to the application of NIR to the heart due to limited penetration through muscle and bone is an important issue to consider. Obvious scenarios would be application of light where needed during cardiac surgery such as coronary artery bypass that carry a significant increased risk of myocardial infarction, or heart transplantation. It might also be possible to apply NIR during a balloon angiography, using a catheter bearing fiber optic through which the light can be delivered to the infarcted area. In addition, authors' unpublished data on dogs demonstrate the feasibility of a transesophageal approach to the heart with a flexible fiber optic NIR probe. The probe in the esophagus or stomach (when advanced) is immediately adjacent to the left atrium and the inferior and posterior walls of the left ventricle. Thus, the anterior wall that is frequently affected by myocardial infarction may be as much as 6 cm away from the probe. Still, it may not be necessary for NIR light to penetrate the area at risk directly. A remote effect of NIR, comparable to remote preconditioning, might still provide protection and lead to a reduction of infarct size. Signaling factors such as heat shock proteins or NO may mediate such effect.

### Conflict of interest statement

The authors declare that the research was conducted in the absence of any commercial or financial relationships that could be construed as a potential conflict of interest.
